# Study on anxiety, depression, and sleep conditions and their interrelations among vocational college students during the COVID-19 pandemic management normalization

**DOI:** 10.3389/fpubh.2024.1385639

**Published:** 2024-11-08

**Authors:** Ru Gao, Hailian Wang, Shan Liu, Xia Wang, Siyuan Song, Yi Wang

**Affiliations:** ^1^The People’s Hospital of Ya’an, Ya’an, Sichuan, China; ^2^Wenjiang District People’s Hospital of Chengdu, Chengdu, Sichuan, China; ^3^Department of Clinical Medicine, Chengdu University of Traditional Chinese Medicine, Chengdu, Sichuan, China; ^4^Clinical Immunology Translational Medicine Key Laboratory of Sichuan Province, Center of Organ Transplantation, Sichuan Academy of Medicine and Sichuan Provincial People’s Hospital, Chengdu, Sichuan, China; ^5^Medical School, Cheng University, Chengdu, Sichuan, China; ^6^Baylor College of Medicine, Houston, TX, United States; ^7^Department of Critical Care Medicine, Clinical Immunology Translational Medicine Key Laboratory of Sichuan Province, Sichuan Academy of Medical Sciences and Sichuan Provincial People’s Hospital, School of Medicine, University of Electronic Science and Technology of China, Chengdu, China

**Keywords:** COVID-19, anxiety, depression, sleep conditions, correlation

## Abstract

**Objective:**

This study investigates the sleep patterns among vocational college students and examines their association with anxiety and depression amidst the ongoing normalization of COVID-19 management strategies.

**Methods:**

In the period of January to February 2022, a comprehensive survey was conducted involving a random sample of 3,300 students. By employing face-to-face interviews, data on general demographics, along with levels of anxiety, depression, and sleep quality, were meticulously gathered and analyzed.

**Results:**

Out of the 3,049 questionnaires deemed valid for analysis, the prevalence rates for anxiety, depression, and insomnia were found to be 9.7, 14.1, and 81.9%, respectively. Through regression analysis, several factors were identified as significant predictors of insomnia: female gender, a self-perceived average or poor family economic status over the last year, moderate psychological stress due to the pandemic and its associated restrictions, extended daily screen time during the pandemic, absence of a routine physical exercise regime, significant disruption or alteration of daily life routines due to the pandemic, presence of anxiety and depression symptoms during the pandemic, and only partial restoration of normal life routines post-pandemic control measures (*p* < 0.001, *p* < 0.005, or *p* < 0.050). A strong correlation was observed among the symptoms of anxiety, depression, and insomnia (all *p* < 0.001). The correlation between stress and depression, depression and insomnia, and anxiety and insomnia were 0.824, 0.714, 0.620, respectively, (all *p* < 0.001).

**Conclusion:**

Given the substantial impact of abrupt or prolonged crisis events, it is imperative to develop and implement specific intervention strategies aimed at safeguarding the psychological well-being of college students.

## Introduction

The COVID-19 pandemic has emerged as a highly stressful life event, precipitating a surge in mental health issues and significant stress-related symptoms across various social demographics (1). Manifestations range from common psychological distresses, such as anxiety and depression, to insomnia and an assortment of physical symptoms (2, 3). The pandemic has not only exacerbated pre-existing mental health conditions but has also been the genesis of new sleep disturbances and the recurrence or onset of mental disorders for some individuals (4, 5). According to WHO reports (6), there has been a 25% increase (approximately 100 million) in the number of anxiety sufferers annually post-COVID-19, with depression cases rising by 60 million each year. Research conducted by Lu Lin’s team (7) on a sample of 56,932 participants from the Chinese general population revealed an uptick in mental health symptoms during the pandemic—depression (27.9%), anxiety (31.6%), insomnia (29.2%), and Post-traumatic Stress Disorder (PTSD) (2 0.4%)—a of which denote higher rates than pre-pandemic levels. The study also noted that individuals diagnosed with or suspected of having COVID-19, along with their close contacts, exhibited a heightened risk for depressive symptoms (8).

The stress associated with COVID-19 has significantly impacted the global prevalence and intensity of anxiety, depression, PTSD, and drug abuse among adolescents (1, 9, 10). Factors such as age, education, geographic location, economic status, and exposure to catastrophic events like COVID-19 play a crucial role in shaping the human response to crises. Students in higher vocational colleges, who are at a critical stage of physical and psychological development, may experience pronounced psychological disturbances and conflicts amidst the pandemic. Coupled with various pressures related to academics, future employment prospects, and social relationships, these stressors may compound, potentially leading to severe mental health challenges, including PTSD (11).

To date, much of the research has focused on the mental health of patients in the early stages of COVID-19, with scant attention paid to the sleep and psychological well-being of students in higher vocational colleges during the pandemic’s normalization phase. This study aims to investigate the sleep patterns and psychological status (e.g., anxiety and depression) of these students within the context of ongoing COVID-19 management efforts, offering theoretical insights for addressing crisis-induced challenges among college students.

## Methods

### Research hypothesis

*Hypothesis 1*: The standardized COVID-19 management measures have a negative relationship with anxiety, depression, and sleep conditions.

*Hypothesis 2*: Anxiety and depression has affected sleep conditions among vocational College Students.

### Study design and participants

The study was conducted by Face-to-face survey from January to February 2022 and obtained confidentiality data. Previous studies have shown the prevalence of mental health problems was 12% in college students (e.g., anxiety or depression), with the type I error probability <0.05, and 1% of the allowable error, in this the minimum sample size was calculated as 2,800.

This study used a multi-stage random cluster sampling method. The sample size was collected in three phases. The one phase, ensured the research area, selecting a district or county for the research area, selected 1 district (Wenjiang) from 12 districts in Chengdu city randomly, using this district as a cluster random sampling unit; the second phase, ensured the study vocational colleges, selected 5 vocational colleges from all of the vocational colleges in Wenjiang district by randomly, using every vocational college as a cluster random sampling unit; the third phase, ensured the research vocational colleges students, select 3,300 students across five vocational colleges in the Wenjiang district of Chengdu city by randomly. Inclusion and exclusion criteria, those who were the students actively enrolled in vocational studies, willingly participated, and completed our survey while residing on campus during the study period were included; those who were staying at home or were under home quarantine were excluded. This study was approved by the ethical committee of Wenjiang District People’s Hospital of Chengdu City (Approval Notice No. 2023–008).

### Data collection instruments

This research gathered data through a comprehensive questionnaire encompassing, general demographic information, affected by COVID-19, lifestyle habits, psychological stress, anxiety, depression, and sleep conditions during the normal management of COVID-19.

### Instrument

The assessment tools utilized in this study encompassed a general demographic questionnaire and three validated scales: the Generalized Anxiety Disorder-7 (GAD-7), the Patient Health Questionnaire-9 (PHQ-9), and the Insomnia Severity Index (ISI) for evaluating sleep quality, anxiety, and depression symptoms, respectively.

### General demographic questionnaire

This tool gathered basic general demographic details, family economic background, parental education levels, and COVID-19-related issues such as isolation, psychological stress, academic and lifestyle changes, physical activity, internet usage, employment concerns, and interpersonal relationships during the pandemic.

### Generalized Anxiety Disorder-7 (GAD-7)

For anxiety assessment, the Generalized Anxiety Disorder-7 (GAD-7) questionnaire was employed (12, 13). GAD-7 scale is a simple and effective assessment tool for the identification of generalized anxiety disorder, which has been widely used abroad, with a sensitivity of 86.8% and a specificity of 93.4% (14). This tool, grounded in DSM-IV criteria, aims to measure the severity and impact of seven anxiety symptoms experienced by individuals in community settings over the preceding 2 weeks (15). The GAD-7 has a scoring system from 0 to 3 for each of its 7 items, totaling the scores 0 to 21, with defined levels of anxiety: no anxiety (from 0 to 4 scores), mild anxiety (5 to 9 scores), moderate anxiety (10 to 14 scores), and severe anxiety (15 scores or above). A score of 10 or more signifies notable anxiety symptoms. In this study, the internal consistency is *α* = 0.966.

### Patient Health Questionnaire-9 (PHQ-9)

Depressive symptoms were assessed using the Patient Health Questionnaire-9 (PHQ-9) (16, 17), which Columbia University developed in the mid-1990s. It is a self-rating scale specifically for the screening of mental disorders in primary health care Settings. It is simpler than other scales and is widely used in the screening of mental disorders in primary health care Settings. The sensitivity and specificity of PHQ-9 > 10 was all 88%, respectively (18). The positive detection rate of PHQ-9 was 31–51% (19), which aligns with DSM-IV criteria for depression. This instrument is designed for the screening of depression in community dwellers over the last 2 weeks, comprising 9 items with scores ranging from 0 to 3, for a total possible score was 0 to 27. The scoring breakdown is as follows: no depression (0–4 scores), mild depression (5–9 scores), moderate depression (10–14 scores), and severe depression (15 or higher scores). A score of 10 or more is indicative of significant depressive symptoms. In this study, the internal consistency is *α* = 0.948.

### Insomnia Severity Index (ISI)

The Insomnia Severity Index (ISI) was used to quantify the severity of recent insomnia symptoms (13) is a self-rating instrument of self-reported insomnia symptoms over the last 2 weeks. It specifically assesses the severity of insomnia. It is widely used in clinical research, and the sensitivity and specificity of ISI were 87 and 75%, respectively (20). This index includes 7 items, each scored from 0 to 4, culminating in a total score of 0–28. The severity of insomnia is categorized as follows: non-significant symptoms (0–7 scores), mild insomnia (8–14 scores), moderate insomnia (15–21 scores), and severe insomnia (22–28 scores). Scores of 8 or more were considered indicative of insomnia symptoms. In this study, the internal consistency is *α* = 0.928.

Data Collection and Quality Assurance Data collectors, consisting of trained doctors and nurses, underwent standardized training and were certified before participation. Surveys were administered in person, where participants were briefed on the study’s objectives and privacy policies. Informed consent was obtained, and participants were all 30 min to complete the survey. Completed questionnaires were collected by designated personnel, and data entry was verified by two individuals to ensure accuracy. Responses not meeting quality standards were excluded from the analysis.

### Statistical analysis

#### Statistical procedures

Descriptive statistics were used to summarize the scale scores, while categorical data were expressed in terms of frequencies and percentages. To compare categorical variables across groups, the chi-square test was applied,

where $\chi^{2}$ is the chi-square test statistic, *O* refers to the observed frequency, and *E* represents the expected frequency under the null hypothesis. Normally distributed variables, the results were presented as mean ± standard deviation. The mean, denoted by μ, to assess factors associated with anxiety, depression, and sleep disturbances, binary logistic regression analysis was performed. The logistic regression function is represented by the *p-*value. The coefficients were estimated through maximum likelihood estimation, with a *p-*value less than 0.05 considered statistically significant.

## Results

### Participant general demographics and the COVID-19 related characteristics

Table 1 delineates the general demographic details and COVID-19-related attributes of the study’s participants. The analysis incorporated data from 3,049 individuals, comprising males 903(29.6%) and females 2,146(70.4%), with a mean age of 20.9 ± 0.02 years. The ethnic comprising Han 2,725(81.9%) and Minority 324(10.6), respectively, a majority of respondents 1718(58.3%) reported living in households of 4–6 people, 2,306(75.6%) respondents the previous year’s family economic situation was average, 2,217(72.7%) respondents not in one-child families, 1,240(40.7%) respondents’ father or mother’s highest education level reported middle school. Delineates 2,793(91.6%) respondents not be quarantined during the normal management of epidemic, 1,304(42.8%) and 246(8.1%) respondents the psychological pressure caused by the epidemic and various restrictions moderately affected and severely affected respectively, a majority of respondents 1836(60.2%) respondents study partial recovery return to normal after the epidemic is controlled, 1718(56.3%) respondents average total internet used 2–≤5 h per day during normal epidemic management (hours), 2099(68.8%) respondents no physical exercise during the COVID-19, 1,671(54.8%) and 238(7.8%) COVID-19 affected the rule of the life moderately affected and severely affected, 1960(64.3%) and 962(31.6%) respondents partial recovery and full-recovery the rule of the life will gradually recover after the epidemic is gradually controlled, 325(10.7%) and 92(3.0%) respondents relationships with family and friends (including Lover) moderately improvements and great improvement, 660(21.6%) and 152(5.0%) compared with before of the COVID-19, epidemic affected the continuing education and job hunting, Moderately affected Severely affected, 660(21.6%) compared with before of the COVID-19, Epidemic affected the Continuing education and job hunting, 295(10.0%) have the anxiety symptoms, 430(14.1%) have the depression symptoms.

**Table 1:** General demographics and the COVID-19 related characteristics (*N* = 3,049).

Variables	*N*(%)
Gender	
Male	903(29.6)
Female	2,146(70.4)
Ethnic	
Han	2,725(89.4)
Minority	324(10.6)
Total number of families living together	
1–3 people	1,096(35.9)
4–6 people	1718(58.3)
More than 7 people	175(5.7)
Self-assessment of family economic income last year	
Very good	107(3.5)
Average	2,306(75.6)
Poor	636(20.9)
One-child families	
Yes	832(27.3)
No	2,217(72.7)
Father or mother’s highest education level	
Primary School or Below	992(32.5)
Middle school	1,240(40.7)
High school or technical secondary school	488(16.0)
College degree and above	329(10.8)
You were be quarantined during the normal management of epidemic	
Yes	256(8.4)
No	2,793(91.6)
The psychological pressure caused by the epidemic and various restrictions	
Little affected	1,499(49.2)
Moderately affected	1,304(42.8)
Severely affected	246(8.1)
Study will return to normal after the epidemic is controlled	
No recovery	86(2.8)
Partial recovery	1836(60.2)
Full-recovery	1,127(37.0)
Average total internet used per day during normal epidemic management (hours)	
≤2 h	738(24.2)
2–≤5 h	1718(56.3)
5–<7 h	484(15.9)
7 h≤	109(3.6)
Physical exercise during the COVID-19	
Yes	950(31.2)
No	2099(68.8)
COVID-19 affected the rule of the life	
Little affected	1,140(37.4)
Moderately affected	1,671(54.8)
Severely affected	238(7.8)
Whether the rule of the life will gradually recover after the epidemic is gradually controlled	
No recovery	127(4.2)
Partial recovery	1960(64.3)
Full-recovery	962(31.6)
Relationships with family and friends (including Lover), compared with before of the COVID-19	
Little deterioration	70(2.3)
Moderately deterioration	358(11.7)
No deterioration	1877(61.6)
Moderately improvements	325(10.7)
Great improvement	92(3.0)
Unclear	223(7.3)
Epidemic affected the continuing education and job hunting	
Little affected	1977(64.8)
Moderately affected	660(21.6)
Severely affected	152(5.0)
Unclear	260(8.5)
If have the anxiety symptoms	
Yes	295(10.0)
No	2,754(90.0)
If have the depression symptoms	
Yes	430(14.1)
No	2,619(85.9)

### The prevalence of insomnia, anxiety, and depressive symptoms

Table 2 delineates the analysis encompassed data from 3,049 respondents. The prevalence of insomnia, anxiety, and depressive symptoms was found to be notably high, with 2,497 participants (81.9%) exhibiting signs of insomnia. Anxiety and depressive symptoms were observed in 295(9.7%) and 430(14.1%) of the participants, respectively.

**Table 2:** Scale scores and prevalence of positive symptoms among higher vocational college students (*N* = 3,049).

Scales	Scores	95%CI	Number of positive symptoms detected (%)
GAD-7	3.28 ± 0.08	3.13–3.45	295 (9.7%)
PHQ-9	4.51 ± 0.10	4.33–4.71	430 (14.1%)
ISI	13.76 ± 0.11	13.53–13.98	2,497 (81.9%)

If the total score of GAD-7 ≥ 10, anxiety symptoms exist; the total score of PHQ-9 ≥ 10 indicates the existence of depressive symptoms; the total ISI score ≥ 8 indicates insomnia.

### Analysis of factors influencing insomnia symptoms

Table 3 delineates the presence of insomnia symptoms was significantly associated with gender, economic status, quarantine experience, psychological stress due to the pandemic, impact on studies and life routine, internet usage, lack of physical activity, and how the pandemic affected further education and employment. Students experiencing anxiety and depressive symptoms also showed a higher incidence of insomnia (*p* < 0.001 or *p* < 0.050).

**Table 3:** Univariate analysis of sleep conditions in higher vocational college students (*N* = 3,049).

Variables	ISI ≥ 8	*X* ^2^
Gender		
Male	695(77.0)	21.03^**^
Female	1802(84.0)	
Ethnic		
Han	2,233(81.9)	0.04
Minority	264(81.5)	
Total number of families living together		
1–3 people	875(79.8)	5.13
4–6 people	1,479(83.2)	
More than 7 people	143(81.7)	
Self-assessment of family economic income last year		
Very good	69(64.5)	22.7^**^
Average	1901(82.4)	
Poor	527(82.9)	
One-child families		
Yes	653(78.5)	8.98^*^
No	1844(83.2)	
Father or mother’s highest education level		
Primary School or Below	808(81.5)	1.44
Middle school	1,023(82.5)	
High school or technical secondary school	403(82.6)	
College degree and above	263(79.9)	
You were be quarantined during the normal management of epidemic		
Yes	225(87.9)	6.77 ^*^
No	2,272(81.3)	
The psychological pressure caused by the epidemic and various restrictions		
Little affected	1,135(75.7)	75.94 ^**^
Moderately affected	1,145(87.8)	
Severely affected	217(88.2)	
Study will return to normal after the epidemic is controlled		
No recovery	71(82.6)	34.50^**^
Partial recovery	1,563(85.1)	
Full-recovery	863(76.6)	
Average total internet used per day during normal epidemic management (hours)		
≤2 h	551(22.1)	36.78^**^
2–≤5 h	1,435(57.5)	
5–<7 h	415(16.6)	
7 h≤	96(3.8)	
Physical exercise during the COVID-19		
Yes	722(76.0)	32.35^**^
No	1775(84.6)	
COVID-19 affected the rule of the life		
Little affected	849(74.5)	68.64 ^**^
Moderately affected	1,437(86.0)	
Severely affected	211(88.7)	
Whether the rule of the life will gradually recover after the epidemic is gradually controlled		
No recovery	99(78.0)	47.49^**^
Partial recovery	1,675(85.5)	
Full-recovery	723(75.2)	
Relationships with family and friends (including Lover), compared with before of the COVID-19		
Little deterioration	57(81.4)	8.12
Moderately deterioration	298(83.2)	
No deterioration	1,544(82.3)	
Moderately improvements	383(82.9)	
Great improvement	76(73.1)	
Unclear	139(78.1)	
Epidemic affected the continuing education and job hunting		
Little affected	1,595(80.7)	15.52^*^
Moderately affected	574(87.0)	
Severely affected	124(81.6)	
Unclear	204(78.5)	
If have the anxiety symptoms		
Yes	288(97.6)	54.51^**^
No	2,209(80.2)	
If have the depression symptoms		
Yes	421(97.9)	86.56^**^
No	2076(79.3)	

^*^P < 0.05, ^**^P < 0.001.

### Regression analysis of insomnia risk factors

Table 4 delineates the regression analysis highlighted several risk factors for insomnia: female gender (OR = 1.34, 95%CI [1.08–1.66], *p* < 0.050), below-average family economic status (OR = 2.16, 95%CI [1.36–3.41], *p* < 0.005) or very poor (OR = 1.90, 95%CI [1.16–3.14], *p* < 0.050), moderate psychological stress from pandemic restrictions (OR = 1.77, 95%CI [1.43–2.21], *p* < 0.001), and increased daily internet usage during the pandemic. Conversely, not being isolated during the pandemic emerged as a protective factor against insomnia (OR = 0.64, 95% CI [0.42–0.96], *p* < 0.050).

**Table 4:** Binary logistic regression analysis of sleep conditions among higher vocational college students (*N* = 3,049).

Variables		*β*	SE	Wald *χ*^2^	*P*	OR	95% CI
Gender	Female	0.29	0.11	7.06	<0.050	1.34	1.08–1.66
Self-assessment of family economic income last year							
	Average	0.77	0.23	10.78	<0.005	2.16	1.36–3.41
	Poor	0.64	0.26	6.38	<0.050	1.90	1.16–3.14
You were be quarantined during the normal management of epidemic	No	−0.45	0.21	4.75	<0.050	0.64	0.42–0.96
The psychological pressure caused by the epidemic and various restrictions	Moderately affected	0.57	0.11	26.46	<0.001	1.77	1.43–2.21
	Severely affected	0.42	0.22	3.55	0.059	1.53	0.98–2.37
Average total internet used per day during normal epidemic management (hours)		0.37	0.11	10.59	<0.005	1.45	1.16–1.81
	2–≤5 h	0.50	0.17	9.16	<0.005	1.65	1.19–2.27
	5–<7 h	0.70	0.33	4.49	<0.050	2.01	1.05–3.84
	7 h≤						
Physical exercise during the COVID-19	No	0.29	0.11	7.04	<0.050	1.34	1.08–1.65
COVID-19 affected the rule of the life	Moderately affected	0.46	0.11	17.62	<0.001	1.59	1.28–1.97
	Severely affected	0.47	0.24	3.87	<0.050	1.60	1.00–2.55
Whether the rule of the life will gradually recover after the COVID-19 is gradually controlled	Partial recovery	0.55	0.27	4.26	<0.050	1.74	1.03–2.93
	Full-recovery	0.33	0.28	1.37	0.242	1.39	0.80–2.39
If have the anxiety symptoms	Yes	0.92	0.45	4.118	<0.050	2.51	1.03–6.08
If have the depression symptoms	Yes	2.01	0.39	26.688	<0.001	7.49	3.49–16.07

### Correlation between anxiety, depression, and sleep quality

Table 5 delineates Pearson correlation analysis revealed a strong association between insomnia and both anxiety and depression symptoms. The correlation coefficient between anxiety and depression was 0.824 (*p* < 0.001), between depression and insomnia was 0.714 (*p* < 0.001), and between anxiety and insomnia was 0.620 (*p* < 0.001), indicating significant interrelations among these psychological and sleep-related symptoms.

**Table 5:** Correlation analysis between anxiety, depression, and sleep conditions in higher vocational college students (*N* = 3,049).

	GAD-7 total scores	PHQ-9 total scores	ISI total scores
GAD-7 total scores	1		
PHQ-9 total scores	0.824^**^	1	
ISI total scores	0.620^**^	0.714^**^	1

**P < 0.001. GAD-7, Generalized Anxiety Disorde-7; PHQ-9, Patient Health Questionnaire-9; ISI, Insomnia Severity Index.

## Discussion

This study focuses on examining the mental health and sleep status of vocational college students in Sichuan, China, during the ongoing COVID-19 pandemic’s normalization phase, identifying key factors influencing their psychological well-being. Maslow’s hierarchy of needs theory, sleep is one of the most basic physiological needs of human beings, it occupies nearly one-third of the time of human life (21, 22). Sleep is the basis for maintaining normal physiological functions and social activities of the human body (23). At the same time, sleep is also closely related to individual emotions, memory, and other psychological activities (24). Whether to ensure adequate and high-quality sleep is often related to the physical and mental health and living state of individuals. However, with the rapid development of science and technology and the continuous improvement of living standards, people are increasingly troubled by various sleep problems while the pace of life is accelerating. Coupled with the impact of the epidemic, during the normalization management period, a series of negative effects on the study, lifestyles, and employment of higher vocational college students have increased the negative impact on sleep status (25). Our research underscores the significant effects various determinants have on students’ anxiety depression and sleep patterns, advocating for customized psychosocial strategies to improve their overall well-being. In educating students through the pandemic, acknowledging the substantial stress and potential psychological impact infectious disease outbreaks can exert is crucial. Experiencing negative emotions during such times is natural and should not lead to excessive mental strain. Acceptance of these feelings is vital for timely mental adjustment and proactive engagement with the pandemic. Rational processing of epidemic information, understanding the virus’s nature, staying informed about the pandemic without succumbing to misinformation, and maintaining trust in governmental public health measures can transform anxiety into constructive action. Ensuring personal protection without undue worry, maintaining open communication channels, and leveraging technology for social interactions can mitigate feelings of isolation. Upholding a healthy lifestyle, including adhering to regular eating and sleeping schedules, helps sustain normalcy. Additionally, establishing sound hygiene practices and mastering stress management and emotional release techniques are essential for psychological resilience.

### The prevalence of anxiety, depression, and insomnia in vocational college students during normal epidemic management

This investigation reveals that a significant portion of vocational college students in Chengdu, Sichuan Province, are experiencing heightened levels of anxiety, depression, and insomnia (9.7, 14.1, and 81.9%, respectively) during the COVID-19 normalization phase. These figures surpass pre-pandemic benchmarks and align with findings from studies on college students’ sleep, anxiety, and depression across various provinces and cities (16, 17, 26). In a period of COVID-19 normalization, research involving 1,676 college students in China, identified an 11.7% rate of emotional distress, with anxiety and depression prevalence at 9.4 and 27.7%, respectively (26). Another study assessed 1,237 students and found a 19.3% rate of psychological abnormalities (16). A previous study observed an exacerbated mental health symptom among students’ dysplasia and normalized phases of the pandemic, likely due to acute stress responses to the sudden outbreak (17) be quarantined, social interactions, academic routines, job prospects, and family dynamics all affected by the COVID-19 have contributed to students’ negative mood states, fostering anxiety, depression, and sleep issues (27, 28). Conversely, some reports suggest an improvement in students’ mental health during the pandemic’s normalization, potentially attributed to post-traumatic growth (11).

### Gender, family economic income affected the insomnia in vocational college students during normal epidemic management

Our findings indicate that males, individuals from higher-income families, those experiencing lesser pandemic-induced psychological stress, and participants engaging in limited online activities reported fewer symptoms of anxiety, depression, and insomnia. Previous studies have found that female students in higher vocational colleges have more sleep disturbances than male students, which may be related to the physical and mental characteristics of female students (29). Generally, female students are more sensitive than male students and are more susceptible to external influences, resulting in emotional fluctuations that affect sleep (30). Too much anxiety about the future may also be a factor leading to the decline of sleep quality in females (31). Basically, consistent with this study, during the normalization management of the epidemic, study, life and employment are all greatly affected. Faced with this negative life event, the sleep status of female is more affected than that of male, which suggests that we should intervene the sleep status of college students. Targeted guidance and intervention should be carried out according to the different characteristics of male and female. These observations are consistent with prior studies, which have highlighted how crisis events differentially impact students based on gender, with concerns about academics, employment, and social relations being prevalent (32). The socioeconomic repercussions of COVID-19 have significantly impacted some students’ family finances, with severe cases leading to bankruptcy (19). Moreover, family health issues have directly influenced students’ mental well-being, contributing to anxiety, depression, and insomnia. A stable family economic condition has been shown to mitigate the psychological impacts of the pandemic (33). The shift toward online learning and isolation has increased screen time and reduced physical activity among students, correlating with deteriorating mental health and sleep quality (34).

### Physical exercise and maintaining a regular routine affected the depression and insomnia in vocational college students during normal epidemic management

Physical exercise and maintaining a regular routine during and post-pandemic have been linked to lower rates of depression and insomnia, corroborating findings from both domestic and international studies (35, 36). Regular physical activity has been found beneficial for enhancing self-efficacy and psychological resilience, improving mental health outcomes during the pandemic (37). However, anxiety and insomnia symptoms have worsened, although depression rates remained unchanged, as shown in studies examining the sleep patterns of over 2,000 students before and during the pandemic (38). Some Australian scholars also support the association between sleep quality and increased anxiety and depression among students and adults (35), likely due to the disruptions caused by COVID-19 restrictions.

### Average total internet used per day, the psychological pressure caused by the epidemic and various restrictions affected the insomnia in vocational college students during normal epidemic management

Previous studies have shown that higher vocational college students who spend more time online on average every day are more likely to have insomnia symptoms (39). Long-term mobile phone use can lead to eye fatigue, vision loss, neck and shoulder pain, and lack of sleep (40). As college students use mobile phones for a longer time, they are more likely to maintain the same posture for a longer time, leading to longer sedentary time. At the same time, the average daily use collection time is long, which has an impact on the interpersonal communication of higher vocational college students, deepening individual loneliness and anxiety, and thus affecting the emotional function of college students (41). Basically, consistent with this study, during the normalization management of the epidemic, because of restrictions on travel, as a result, the average daily mobile phone use time increased. Previous studies have shown that sleep status is significantly negatively associated with anxiety and depression in college students in China, and the worse the sleep status, the higher the risk of anxiety and depression symptoms. Studies have shown that the basic rhythm of life including sleep is an important manifestation of the body’s adapt to the environment (42, 43). Previous studies have found that psychological problems are negatively correlated with regular living habits, and college students with anxiety and depression have more irregular living habits such as diet and sleep (44). A good level of mental health is conducive to college students to have a clear understanding of themselves and their environment, reasonable arrangement of work, study, and life, and effective implementation. The increase in anxiety or depression may have an impact on diet, sleep, and other behaviors in daily life, affecting physical and mental health. At the same time, some studies have shown that regular life can reduce the incidence of depression and relieve negative emotions, which suggests that college students should arrange their daily life reasonably and regularly study, sleep, diet, and exercise when facing the pressure brought by the epidemic (45, 46). During the early epidemic period of COVID-19, anxiety and depression symptoms of Chinese college students increased compared with normal levels, which was consistent with the results of Chinese scholars’ investigation of the psychological status of some college students during SARS in 2003 (47). Foreign scholars’ investigation of college students in the early epidemic period found that in the early stage of the COVID-19 global pandemic, The anxiety and depression symptoms of students in universities and colleges in different countries have increased compared with those before the epidemic, and their psychological stress has increased (48). Therefore, adequate and good sleep helps to maintain and regulate the good operation of the body’s neuroendocrine system and improve the body’s emotional regulation ability and emotional stability. When there are problems in sleep conditions, such as sleep quality decline, sleep late, and lack of sleep, it is easy to lead to endocrine disorders of the body, so people’s emotions are more likely to fluctuate. It increases the sensitivity of the body to external pressure and promotes the occurrence of psychological stress symptoms (49).

At the same time, studies have found that the psychological stress of college students is also one of the predictors of sleep status, that is, the higher the stress level, the worse the sleep quality of college students. This may be because when the pressure load is too large, it is easy to cause excessive emotional fluctuations in the body, leading to depression, anxiety, and other negative emotions, which hurt sleep quality (36). In mild cases, it will cause difficulty in falling asleep, light sleep, and shorter sleep time, and in severe cases, it will even lead to sleep disorders. In addition, the research results of Van Someren et al. (50) showed that when people are in a state of stress, certain stress-related hormones are produced in the body, leading to increased secretion of cortical hormones and cortisol, which causes brain wave disorder and even contributes to the occurrence of insomnia. However, the deterioration of sleep may further aggravate the symptoms of psychological stress, thus falling into a vicious cycle. This suggests that there may be a bidirectional relationship between sleep status and psychological stress levels in college students. Compared with undergraduates, vocational students have to complete the heavy learning tasks of “combining work with study” and face increasingly fierce employment competition. Healthy sleep helps vocational students to efficiently complete theoretical learning and skill training. Therefore, we can see that higher vocational college students are facing pressure from work, employment, life, and other pressures, coupled with the normalization of the epidemic management, which brings new pressure to employment, family economic status, education, interpersonal communication, etc. The superposition of the two pressures can easily lead to anxiety, depression, and other negative emotions, and even other psychological problems, thus affecting sleep status.

### The anxiety and depression symptoms affected insomnia in vocational college students

Previous studies have shown that there is a correlation between insomnia and anxiety and depression, and sleep is easily disturbed by depression and anxiety (22, 51). At the same time, anxiety and depression can cause or aggravate sleep disorders. Kim et al.’s (52) study on sleep status in Korean universities showed that insomnia symptoms were correlated with anxiety and depression. College students are more likely to suffer from mental health problems such as depression, anxiety, and stress symptoms than other groups, usually due to changes in social roles and reduced social support. Many studies have shown that the number, frequency, and severity of mental health problems among college students have increased in recent years, which indicates that college students generally have different degrees of psychological disorders and psychological distress (53). A study based on nearly 20,000 freshmen found that although the mental health of college students is generally improving, it is still not optimistic (54). Negative emotional and mood problems, including depression, anxiety, and stress, are common and tend to be younger. In addition, problems such as mental toughness, environmental adaptability, and interpersonal relationship processing ability are also relatively deficient. Some foreign studies show that 12–50% of college students have met the criteria for one or even more common mental disorders (55). There are also many reports on the research of college students’ mental health problems in China. The results of the sample survey of college students organized by the former State Education Commission show that 20.13% of college students have psychological problems, especially in recent years, with the increase of the only child in college students, psychological problems and even mental disorders are more prominent (56). The above research shows that the mental health problems of college students are no longer an individual phenomenon, but a certain universality in a certain range, and begin to become a prominent representative problem in the university, which must be paid enough attention to and take appropriate measures to regulate. Especially during the normalization management of the epidemic, the superposition of the adverse effects of the epidemic will lead to the aggravation of their anxiety and depression symptoms, which will affect their sleep status. There is a two-way relationship between adverse psychological emotions and sleep status, and a series of adverse effects will adversely affect the development and progress of higher vocational college students. Even endanger the happiness of the family and social harmony and stability.

### Limitations

This study, being cross-sectional, lacks longitudinal data to establish causality between psychological issues, sleep disturbances, and identified risk factors. Moreover, it relies on self-reported measures of anxiety, depression, and insomnia, which may not align with clinical assessments. Furthermore, the applicability of these findings may be limited by geographic, cultural, and economic variations.

## Conclusion

In conclusion, the ongoing management of the pandemic and associated restrictions necessitate addressing the compounded pressures faced by vocational college students, including academic, employment, and financial stresses. Conducted 2 years post-outbreak, this study provides valuable insights into the mental health and sleep patterns of vocational college students in Chengdu, Sichuan Province, contributing to the broader understanding of pandemic-induced psychological impacts and offering a basis for emergency psychological interventions and stress responses among college students.

## Data Availability

The original contributions presented in the study are included in the article/supplementary material, further inquiries can be directed to the corresponding authors.
